# High-throughput surface epitope immunoaffinity isolation of extracellular vesicles and downstream analysis

**DOI:** 10.1093/biomethods/bpae032

**Published:** 2024-05-17

**Authors:** Ramin Khanabdali, Michelle Mandrekar, Rick Grygiel, Phuoc-An Vo, Carlos Palma, Sara Nikseresht, Siena Barton, Mozhgan Shojaee, Sadman Bhuiyan, Kartini Asari, Susan Belzer, Khairul Ansari, Jermaine I Coward, Lewis Perrin, John Hooper, Dominic Guanzon, Andrew Lai, Carlos Salomon, Kevin Kershner, Christine Newton, Douglas Horejsh, Gregory Rice

**Affiliations:** INOVIQ Ltd., Notting Hill, VIC 3168, Australia; Promega Corporation, Madison, WI 53711, United States; Promega Corporation, Madison, WI 53711, United States; Promega Corporation, Madison, WI 53711, United States; INOVIQ Ltd., Notting Hill, VIC 3168, Australia; INOVIQ Ltd., Notting Hill, VIC 3168, Australia; INOVIQ Ltd., Notting Hill, VIC 3168, Australia; INOVIQ Ltd., Notting Hill, VIC 3168, Australia; INOVIQ Ltd., Notting Hill, VIC 3168, Australia; INOVIQ Ltd., Notting Hill, VIC 3168, Australia; INOVIQ Ltd., Notting Hill, VIC 3168, Australia; INOVIQ Ltd., Notting Hill, VIC 3168, Australia; Mater Research Institute, The University of Queensland, Translational Research Institute, Woolloongabba, QLD 4102, Australia; ICON Cancer Care, South Brisbane, QLD 4101, Australia; Mater Research Institute, The University of Queensland, Translational Research Institute, Woolloongabba, QLD 4102, Australia; Mater Research Institute, The University of Queensland, Translational Research Institute, Woolloongabba, QLD 4102, Australia; Translational Extracellular Vesicles in Obstetrics and Gynae-Oncology Group, UQ Centre for Clinical Research, Royal Brisbane and Women’s Hospital, Faculty of Medicine, The University of Queensland, Brisbane, Australia; Translational Extracellular Vesicles in Obstetrics and Gynae-Oncology Group, UQ Centre for Clinical Research, Royal Brisbane and Women’s Hospital, Faculty of Medicine, The University of Queensland, Brisbane, Australia; Translational Extracellular Vesicles in Obstetrics and Gynae-Oncology Group, UQ Centre for Clinical Research, Royal Brisbane and Women’s Hospital, Faculty of Medicine, The University of Queensland, Brisbane, Australia; Promega Corporation, Madison, WI 53711, United States; Promega Corporation, Madison, WI 53711, United States; Promega Corporation, Madison, WI 53711, United States; INOVIQ Ltd., Notting Hill, VIC 3168, Australia; Translational Extracellular Vesicles in Obstetrics and Gynae-Oncology Group, UQ Centre for Clinical Research, Royal Brisbane and Women’s Hospital, Faculty of Medicine, The University of Queensland, Brisbane, Australia

**Keywords:** Extracellular vesicles (EVs), Exosomes, High-throughput, Immunoaffinity, EV RNA and protein isolation

## Abstract

Extracellular vesicles (EVs), including exosomes, have significant potential for diagnostic and therapeutic applications. The lack of standardized methods for efficient and high-throughput isolation and analysis of EVs, however, has limited their widespread use in clinical practice. Surface epitope immunoaffinity (SEI) isolation utilizes affinity ligands, including antibodies, aptamers, or lectins, that target specific surface proteins present on EVs. Paramagnetic bead-SEI isolation represents a fit-for-purpose solution for the reproducible, high-throughput isolation of EVs from biofluids and downstream analysis of RNA, protein, and lipid biomarkers that is compatible with clinical laboratory workflows. This study evaluates a new SEI isolation method for enriching subpopulations of EVs. EVs were isolated from human plasma using a bead-based SEI method designed for on-bead and downstream analysis of EV-associated RNA and protein biomarkers. Western blot analysis confirmed the presence of EV markers in the captured nanoparticles. Mass spectrometry analysis of the SEI lysate identified over 1500 proteins, with the top 100 including known EV-associated proteins. microRNA (miRNA) sequencing followed by RT-qPCR analysis identified EV-associated miRNA transcripts. Using SEI, EVs were isolated using automated high-throughput particle moving instruments, demonstrating equal or higher protein and miRNA yield and recovery compared to manual processing. SEI is a rapid, efficient, and high-throughput method for isolating enriched populations of EVs; effectively reducing contamination and enabling the isolation of a specific subpopulation of EVs. In this study, high-throughput EV isolation and RNA extraction have been successfully implemented. This technology holds great promise for advancing the field of EV research and facilitating their application for biomarker discovery and clinical research.

## Introduction

The release of lipid membrane-bound extracellular vesicles (EVs) by cells into extracellular fluids contributes to the maintenance of cellular homoeostasis [1–3], inter-cellular communication [4–7], and pathogenesis [8–10]. The concentration, composition, and biological activity of EVs present in biofluids [11–15], such as blood [16–18], saliva [19, 20], and urine [21, 22] are altered in response to changes in physiological status [15, 23], and disease onset and progression [8, 24]. The molecular phenotype of such EVs has been reported to be informative of the parent cell phenotype [25–28] and affords opportunity for non-invasive assessment of parent cell status and identification of cell-specific biomarkers that may be useful in the earlier diagnosis of many diseases [7]. Although the compendium of identified disease-associated EV biomarkers continues to increase [29–31], the translation of EV-based diagnostics into routine clinical laboratory tests, such as Laboratory Developed Tests implemented in Clinical Laboratory Improvement Amendments (CLIA)-certified laboratories [32], remains limited. The challenge lies not in establishing and verifying the analytical and clinical performance of EV-based tests, as defined by FDA 42 Code of Federal Regulation (CFR) 493.1253, but rather in the availability of simple, rapid, reproducible, high-throughput EV isolation, and downstream analysis methods that are compatible with clinical laboratory workflows.

EV isolation methods based on physicochemical properties (e.g. particle density, size, or charge) are confounded by the co-isolation of substances that share similar characteristics [33]. The resultant miscellany may compromise downstream analytical and functional investigations and the deconvolution of EV and non-EV contributions [34].

One approach that offers improved specificity in isolating EV subpopulations is surface epitope immunoaffinity (SEI) isolation. This method isolates EV subpopulations defined by biosynthetic processes imprinted by their parent cell [7]. SEI isolation utilizes affinity ligands (e.g. antibodies, aptamers, or lectins) that target specific surface proteins present on EVs. Combining SEI capture with paramagnetic beads provides a solution for the reproducible, high-throughput isolation of EVs from biofluids and downstream analysis of RNA, protein, and lipid biomarkers that is compatible with clinical laboratory workflows. Such methods are compatible with on-bead analysis (e.g. Fourier Transformed InfraRed Spectroscopy [35, 36], ELISA [36, 37], and flow cytometry [38]) or on-bead lysis and downstream analysis (e.g. mRNA or microRNA (miRNA) seq and mass spectrometry (MS) [39]). The development of such high-throughput EV isolation methods will enable the translation of EV biomarkers into clinically relevant liquid biopsies.

The aim of this study was to evaluate a new high-throughput SEI method for isolating EVs and analysing their protein and RNA cargo (EXO-NET^®^ Inoviq, Melbourne, Australia). EXO-NET is a molecular net (USA patent US20140080119A1) composed of 10 antibodies against integral membrane proteins assembled on a paramagnetic bead. The sequential construction of the 3D antibody matrix increases the stability, density, and accessibility of ligand binding sites. The isolation tool was developed to capture EVs for on-bead-analysis or on-bead-lysis and downstream analysis of the associated cargo.

Following the guidelines outlined in Minimal Information for Studies of EVs (MISEV) [40], EVs were characterized based on the presence of known EV surface protein markers, including Category 1 transmembrane/GPI-linked proteins (CD9, CD63, and CD81), and Category 2 cytosolic proteins (Flotillin-1 and TSG101). The presence of RNase-resistant RNA (i.e. vesicle-encapsulated RNA) was confirmed by quantifying the relative abundance of miRNAs and mRNAs after RNase A treatment in the presence and absence of a membrane solubilizing detergent. To evaluate compatibility with proteomic and genomic profiling, the molecular cargo of SEI-isolated EVs was profiled using SWATH MS, RT-qPCR, and miRNASeq. Furthermore, using SEI, EVs, and EV-associated protein and RNA were isolated using automated high-throughput particle moving instruments, demonstrating equal or higher protein and miRNA yield and recovery compared to manual processing.

## Materials and methods

### Clinical research samples

Plasma samples were obtained from commercial suppliers (BioIVT, Westbury, NY, USA and ProteoGenex, Inglewood, CA, USA). Plasma samples for MS, miRNASeq, and real-time polymerase chain reaction (RT-qPCR) studies were obtained from the Mater Hospital (Brisbane, QLD, Australia) in accordance with the declaration of Helsinki and approved by the Ethics Committee of The University of Queensland (2020/HE001852). Peripheral blood samples (10 ml) were obtained, with informed consent, from normal healthy women. EDTA anticoagulated plasma was prepared by centrifuging the samples at 2500 *g* for 10 min and 0.5 ml aliquots was stored at −80°C.

### SEI extracellular vesicle isolation

#### Optimization of SEI reagent and input sample volume 

To determine the optimal volumes of SEI reagent and plasma, titration curves of SEI reagent (3.5, 7, 15, 30, and 50 μl) with a fixed volume of pooled human plasma (500 µl), and plasma (160, 320, 640, 1000, and 1500 μl) at a fixed SEI reagent volume (15 µl) were performed in triplicate. EV-associated miRNA was used as a measure of EV isolation. Pooled plasma samples were incubated with SEI reagent for 15 min at room temperature. The beads were immobilized by magnet and washed three times (1 ml Dulbecco’s phosphate buffer saline, DPBS). After the final wash, the beads were suspended in 250 μl of BL buffer supplied with ReliaPrep^TM^ RNA Miniprep Systems for RNA extraction (Promega, Madison, WI, USA; Cat #Z6010). RNA was eluted in 20 μl nuclease-free water and 12 μl was used to perform a reverse transcription reaction using the TaqMan 5× hsa-miR-21-5p primer (Thermo Fisher Scientific, Waltham, MA, USA; Cat #4427975, assay ID 000397) and Reverse Transcription TaqMan miRNA kit (Thermo Fisher Scientific; Cat #4366596). Four microlitres of the cDNA were used for qPCR reaction using TaqMan 20× hsa-miR-21-5p primer (Thermo Fisher Scientific; Cat #4427975, assay ID: 000397). Standard miR21 reactions were run using 1, 10, and 100 fM miR21. Data are summarized as mean CT values ± the standard error of the mean (SEM) (*n* = 3).

#### RNase-resistant EV-associated miRNA

To confirm that RNA captured by the SEI reagent was vesicle-associated, bead-captured EVs were incubated in the presence or absence of RNase A (Thermo Fisher Scientific; Cat #EN0531) (6.25 µg/ml) or RNase A (6.25 µg/ml) and 0.1% Triton X-100. Treated beads were magnetically immobilized and washed, and RNA extracted (as detailed above). The presence of mRNAs (GAPDH and RPLP0) and miRNAs (miR16 and miR21) was established by RT-qPCR.

#### Manual SEI EV isolation

**Figure 1.**
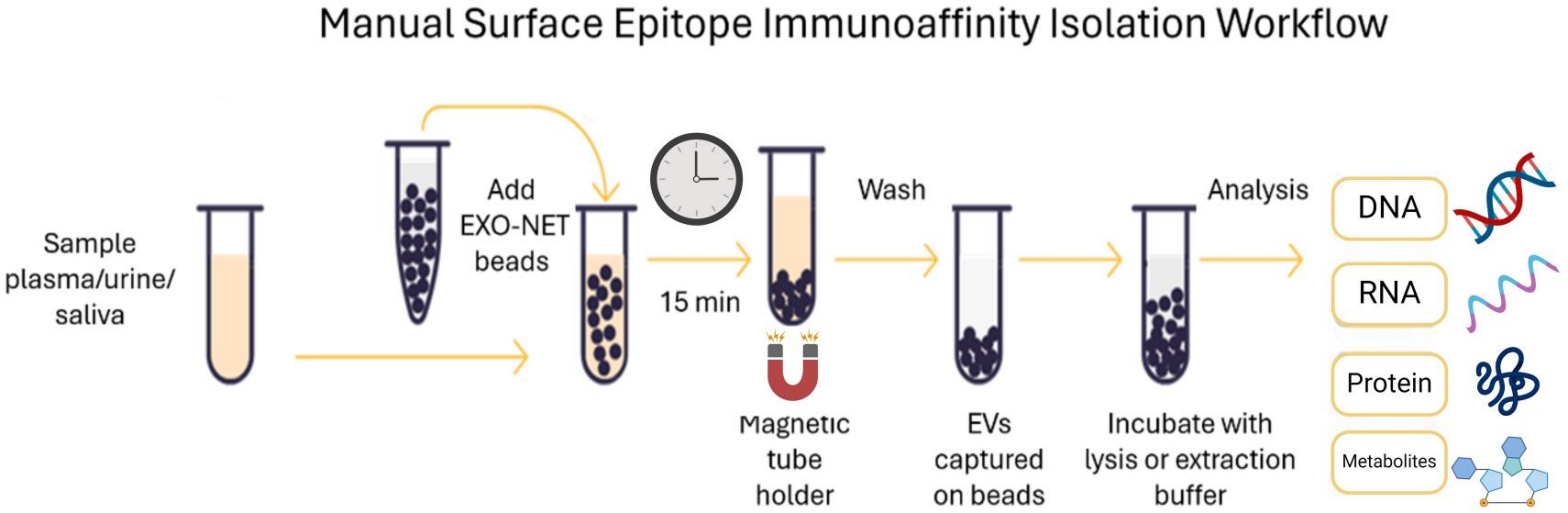
Extracellular vesicle SEI manual isolation workflow.

SEI EV isolations were performed in triplicate using 500 µl of pooled normal human plasma, according to the manufacturer’s instructions (Fig. 1). Plasma samples were thawed at room temperature and subsequently centrifuged for 5 min at 10,000*g*. The clarified plasma (500 µl) was transferred to a new 1.5 ml microfuge tube and 30 µl of beads were added. The tube was gently mixed, incubated for 15 min at room temperature and then placed on a magnetic rack (Thermo Fisher Scientific; Cat #CS15000) for 5 min. The supernatant (unbound material) was removed. The beads were washed three times by resuspension in 1 ml of DPBS and magnet immobilized. Following the final wash, the beads were resuspended in lysis buffer for downstream analyses.

#### Automated high-throughput SEI EV isolation

**Figure 2.**
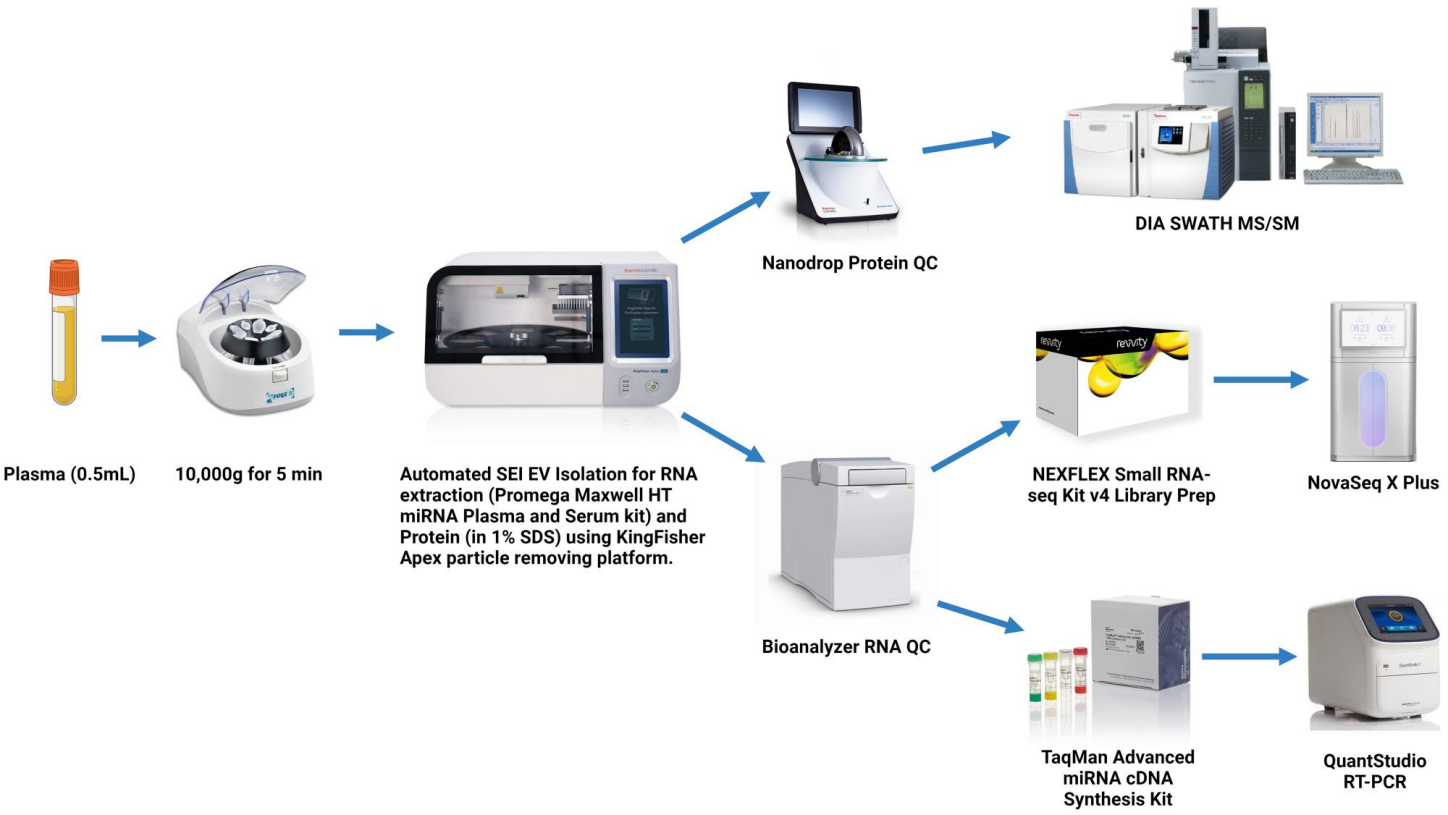
SEI automated workflow for high-throughput EV isolation, RNA, and protein extraction for downstream analysis.

EVs were isolated from normal human plasma (500 µl) using a 96-well plate magnetic particle moving instrument (KingFisher Apex; Thermo Fisher Scientific) (Fig. 2, see also, Supplementary Protocols). Following EV isolation, RNA was extracted using Maxwell^®^ RSC miRNA Plasma and Serum Kit (Promega; Cat #AS1680) according to the manufacturer’s instructions. For Western Blot and MS analyses, bead-captured EVs were lysed with 1% SDS. SEI isolation of EVs was also implemented on the Maxwell RSC instrument (Promega) for medium-throughput sample processing (see Supplementary Fig. S1).

#### EV isolation method comparison

EVs were isolated from plasma using five commercially available EV isolation kits: ExoQuick (SBI, Palo Alto, CA, USA), ExoEasy (Qiagen, Ann Arbor, MI, USA), Plasma/Serum Exosome purification (Norgen Biotek, Thorold, Ontario, CA, USA), Total Exosome Isolation Kit (Thermo Fisher Scientific), and SEI (EXO-NET; Inoviq Ltd Melbourne, Australia). Isolations were performed in triplicate according to manufacturers’ instructions.

### Western blot analysis

For the analysis of EV-associated proteins, EV captured by SEI beads were lysed in 30 µl of 1% SDS in ultrapure water (Sigma-Aldrich; Cat #L3771) and incubated for 15 min at room temperature. Protein concentration (2 µl lysate) was quantified by Nanodrop One spectrophotometer (Thermo Fisher Scientific) at an absorbance of 280 nm. Lysate (10–20 µg protein) was mixed with 7.5 µl of 4× Bolt LDS Sample Buffer (Thermo Fisher Scientific; cat #B007) and 3 µl of 10× Bolt Sample Reducing Agent (Thermo Fisher Scientific; Cat #B0009) and incubated at 95°C for 3 min. After denaturation, lysates were resolved on 4–12% Bolt Bis–Tris Plus gels (Thermo Fisher Scientific; Cat #NW04120BOX) and subsequently transferred to iBLOT 2 PVDF regular stack membranes (Thermo Fisher Scientific; Cat #IB23001). The membranes were blocked for 1 h at room temperature using a blocking solution consisting of 2.5% BSA (Sigma; Cat #A9647-50G) prepared in 20× Tris-Buffered Saline (Thermo Fisher Scientific; Cat #28358) with 0.1% Tween (TBS-T). After blocking, the membranes were incubated overnight at 4°C with primary antibodies at a 1:500 dilution in TBS-T + 2.5% BSA. The primary antibodies used were against CD9 (Cell Signalling Technology, Danvers, MA, USA; Cat #13174 T), CD63 (Cell Signalling; Cat #52090), CD81 (Cell Signalling; Cat #528923), TSG101 (Cell Signalling Technology; Cat #72312), and Flotillin-1 (Cell Signalling Technology; Cat #18634 T). Following primary antibody incubation, the membranes were washed with TBS-T and incubated with an HRP-conjugated anti-rabbit IgG secondary antibody (Cell Signalling Technology; Cat #7074P2) at a 1:2000 dilution in TBS-T + 2.5% BSA for 1 h at RT. After another round of washing, the membranes were visualized using the Amersham ECL detection reagent (Cytiva, Marlborough, MA, USA; Cat #17352401) and a ChemiDoc MP imaging system (Bio-Rad, Hercules, CA, USA).

### Quantitative proteomics and SWATH analysis

#### Filter aided sample preparation

For SWATH analysis, individual exosome samples were processed using the Filter Aided Sample Preparation method [26]. A total of 15 µg of exosome protein from each sample was reduced with equal volume of lysis buffer containing 8% SDS, 100 mM Tris, pH 7.6 and 0.2 M DTT, followed by sonication and heating of samples at 95°C. Samples were allowed to cool down completely before adding 8 M urea in 100 mM Tris, pH 8.5. Samples were transferred into a Nanosep^®^ filter unit with a 30K molecular weight cut-off and centrifuged for 10 000 *g* for 15 min. Then, filter units were washed with 400 µl of urea buffer and centrifuged for 10 000 *g* for 15 min. Samples were alkylated by the addition of 100 µl of 50 mM IAA in 8M urea buffer and incubated in the dark for 20 min. The filter units were washed with 8M urea buffer. Proteins were digested using 0.3 µg of trypsin and incubated overnight at 37°C.

#### Desalting

The solubilized peptides from pooled and individual samples were desalted using SOLAµ HRP SPE 96-well plate (Thermo Fisher Scientific) according to the manufacturer’s instruction.

#### Analysis of peptides

Tryptic digest was loaded onto a reversed-phase trap column (CHROMXP C18CL 5 um, 10 × 0.3 mm; Eksigent, Redwood City, CA, USA) and on column wash was performed for 15 min (3 µl/min) followed by peptide separation on reversed phase CHROMXP C18CL 3 um, 120 A0, 150 × 0.075 mm (Eksigent) analytical column. LC gradient started with 95% mobile phase A (H2O/0.1% FA), 5% B (ACN/0.1% FA) at 0 min and increase to 10% B over for 2 min and then a 58 min linear gradient to 40% B followed by 50% B for 5 min. Mobile phase B was then increased from 50% to 95% over 10 min followed by column wash at 95% B for 15 min and re-equilibrated with 5% Buffer B for 6 min. The flow rate was kept at 250 nl/min during the entire LC run. The resulting peptide samples were processed in IDA on an AB Sciex 5600 TripleTOF mass spectrometer with the top 18 precursor ions automatically selected for fragmentation. The data obtained were combined to establish a peptide ion database. For SWATH acquisition, the TripleTOF^®^ 5600 System was configured as described by Gillet *et al.* [27]. Using an isolation width of 26 Da (25 Da of optimal ion transmission efficiency and 1 Da for the window overlap), a set of 32 overlapping windows was constructed covering the mass range of 400–1200 *m/z*. The raw MS data have been deposited at the following location: https://doi.org/10.48610/1958974.

#### Data processing

To generate the local ion library, a protein database search was conducted using the ProteinPilot version 4.5b Software (SCIEX, Framingham, MA, USA) and the Paragon^™^ Algorithm. The search was performed against the SwissProt Homo sapiens database with a global false discovery rate (FDR) of 1% and was used as the threshold for the number of proteins for import. The SWATH Acquisition Microapp version 2.0 in PeakView version 2.2 (SCIEX) was used to create a spectral library file. This local library was extended using the R package SwathXtend (version 2.3) [28] with a published SWATH dataset of healthy human plasma [29]. The extended library was used for all subsequent SWATH analysis. Processing settings for the SWATH Microapp: two peptides per protein, three transitions per peptide, peptide confidence threshold corresponding to 1% global FDR and FDR threshold of 1% was used. The retention time was then manually realigned with a minimum of five peptides with constantly high signal intensities and distributed along the time axis. The resulting peak area for each protein after SWATH processing was exported to MarkerView 1.3.1 (SCIEX) for statistical analysis. The resulting data were normalized using the Total Area Sums approach.

### RNA extraction and qPCR analysis

For total RNA extraction, bead-bound EVs from 500 µl of normal human plasma were incubated for 30 min at room temperature in a 500 µl digestion mixture of either 6.25 µg/ml RNase A and/or 0.3% Triton X-100 in PBS. The RNA was then extracted using ReliaPrep^™^ RNA Miniprep Systems (Promega; Cat #Z6012) according to the manufacturer’s instructions. To quantify mRNA, reverse transcription was performed using the SuperScript VILO cDNA Synthesis Kit (Thermo Fisher Scientific; Cat #11754050). The specific mRNA targets analysed included GAPDH (Thermo Fisher Scientific; Cat #4331182, Assay ID: Hs03929097_g1), RPLP0 (Thermo Fisher Scientific; Cat #4331182, Assay ID: Hs00420895_gH), OAZ1 (Thermo Fisher Scientific; Cat #4331182, Assay ID: Hs00427923_m1), and SERF2 (Thermo Fisher Scientific; Cat #4331182, Assay ID: Hs00428481_m1). To quantify miRNA, reverse transcription was performed using the TaqMan^™^ MicroRNA Reverse Transcription Kit (Thermo Fisher Scientific; Cat #4366596) following the manufacturer’s instructions. The specific miRNA targets analysed included miR16 (Thermo Fisher Scientific; Cat #4427975, Assay ID: 000391), miR21 (Thermo Fisher Scientific; Cat #4427975, Assay ID: 000397), and let7a (Thermo Fisher Scientific; Cat #4427975, Assay ID: 000377). Quantitative PCR (qPCR) was performed using the QuantStudio^™^ 5 Real-Time PCR System (Thermo Fisher Scientific) with the following reaction conditions: an initial denaturation at 95°C for 20 s, followed by 40 cycles of denaturation at 95°C for 15 s and annealing/extension at 60°C for 60 s. A CT threshold of 0.1 was set for all the assays, and the data were analysed in QuantStudio 5 Design and Analysis Software version 1.5.2.

For the automated EV and miRNA experiments, miRNA quantitation was performed with the TaqMan^™^ MicroRNA Reverse Transcription Kit (Thermo Fisher Scientific; Cat# 4366596) and the GoTaq^®^ Probe qPCR Master Mix (Promega; Cat #A6102), following the manufacturer’s instructions. The Veriti Thermal Cycler (Thermo Fisher Scientific) was used for reverse transcription of miRNA and the Applied Biosystems 7500 Fast Real-Time PCR (Thermo Fisher Scientific) qPCR was used for qPCR after reverse transcription. Quantitation of mRNA was performed using GoTaq^®^ Probe 1-Step RT-qPCR (Promega; Cat #A6120), following the manufacturer’s instructions, with the 7500 Fast Real-Time Instrument or QuantStudio^™^ 6 Pro (Thermo Fisher Scientific) with Design and Analysis Software version 2.6.0.

### RNA next-generation sequencing

The RNA from EXO-NET captured EVs were extracted using the Exosomal RNA Isolation Kit (Norgen Biotek; Cat #58000) following the manufacturer’s instruction, with the modification that EXO-NET beads were magnetically separated from the lysate after 10 min lysis incubation. Subsequently, the RNA was quantified using the Qubit^™^ microRNA Assay Kit (Thermo Fisher Scientific). A total of 1–2 ng of EV RNA was used as input for the NEXTFLEX^®^ Small RNA-seq v3 with UDI barcodes (PerkinElmer, Shelton, CT, USA) to generate sequencing libraries, according to the manufacturer’s instructions for low input, with the following modifications. The adaptors were diluted by 1/4th with H20 for ligation reactions, PCR was performed for 25 cycles and libraries were eluted from size-selected gel pieces overnight with shaking. The final library was quantified using the KAPA Library Quantification Kit (Roche, Palo Alto, CA, USA) and library size was determined using the Tapestation High Sensitivity D1000. A total of 96 samples were pooled in equimolar quantities, and the final pooled library was sequenced using the NextSeq 500 and 75 cycles High Output kit (single end for 75 cycles).

### Identification, normalization, statistical, and gene ontology analysis of microRNAs

Initially, raw FASTQ files were processed to remove adaptor sequences and random bases using Cutadapt, software with parameters recommended by PerkinElmer for the NEXTFLEX^®^ Small RNA-seq version 3. Subsequently, this file was analysed using the miRDeep2 program to identify known miRNAs [41]. The miRDeep2 algorithm requires a genomic index and microRNA database to perform analysis. The human genome (GRCh38) pre-built index was obtained from the bowtie website (http://bowtie-bio.sourceforge.net/index.shtml) [42]. The miRNA reference database (version 22.1) was obtained from the miRBase website (http://www.mirbase.org/) [43]. Subsequently, raw counts and corresponding microRNAs underwent normalization and statistical analysis using the DESeq2 package in R [44]. This package uses the median ratio method for normalization and applies a generalized linear model for differential expression. Furthermore, statistical analysis and significance were calculated using a Wald test [45], corrected for multiple testing using the Benjamini and Hochberg procedure [46]. Finally, the microRNAs were subjected to over-representation gene ontology analysis using miEAA version 2.0 software [47].

### Data collection and statistical analysis

MS and miRNA Seq analysis were conducted within an ISO17025 accredited (National Association of Testing Authorities, Australia) research facility at Translational EVs in Obstetrics and Gynae-Oncology Group. All data were recorded within a 21 CFR part 11 compliant electronic laboratory notebook (Lab Archives, Carlsbad, CA, USA). Data were analysed using GraphPad Prism version 8 (Graphpad, San Diego, CA, USA). All data are presented either as mean ± SEM unless otherwise indicated or as representative single experiments. A *P* > 0.05 was ascribed as not significant (ns). Statistically significant difference was ascribed as *P* ≤.05 *, ≤.01 **, ≤.001 ***, and ≤.0001 ****.

## Results

### Optimization of SEI reagent and input sample

**Figure 3.**
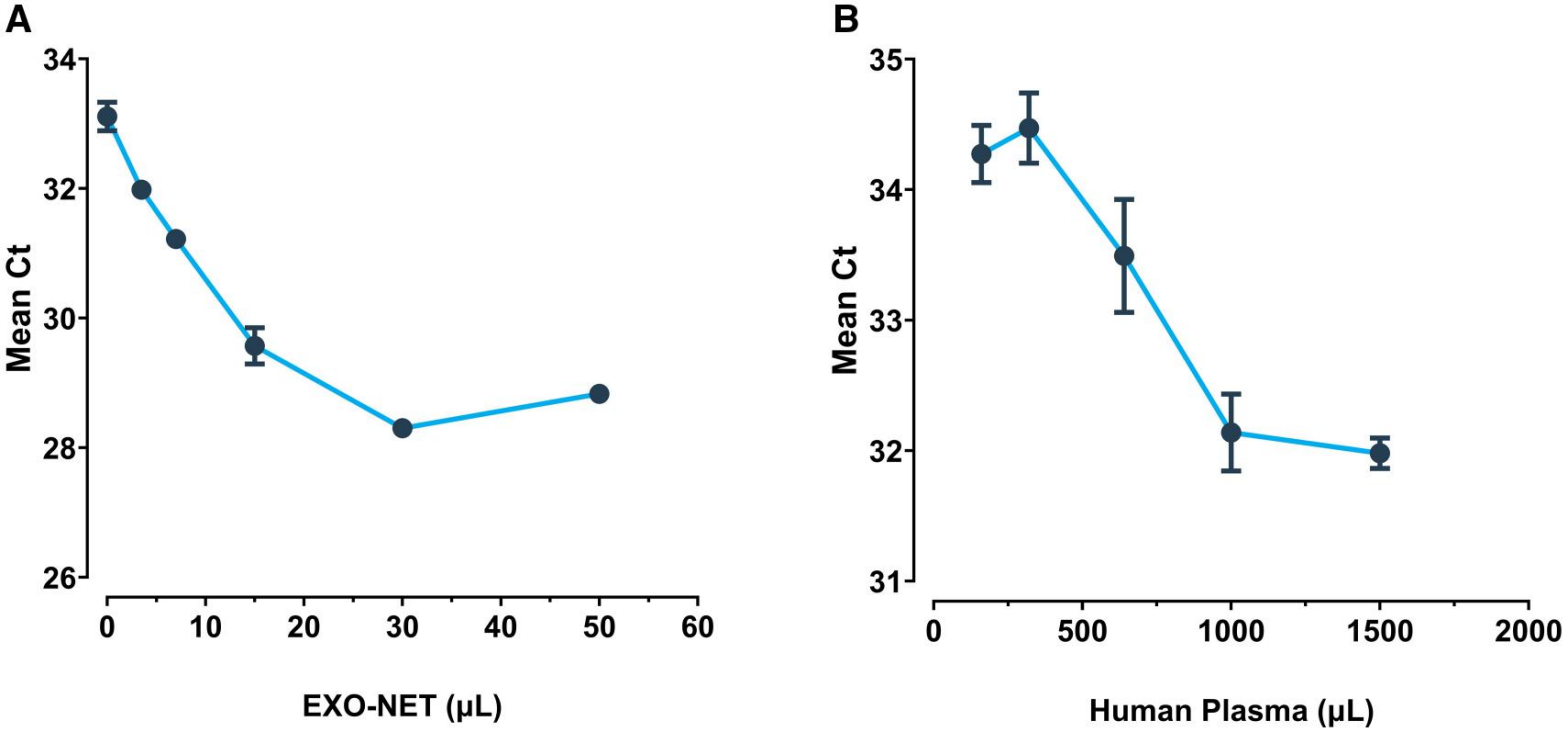
Optimization of SEI reagent and plasma volume. Data are presented as mean miR21 Ct ± SEM (*n* = 3).

miR21 Ct value titration curves for SEI reagent (A) and input pooled plasma sample volume are presented in (Fig. 3). Mean Ct values for EV-associated miR21 are linearly repeated to both SEI and sample input volume. As a set plasma sample input volume of 500 µl, mean miR21 Ct values decrease with increasing SEI reagent volume up to 30 µl. At a set SEI reagent volume of 15 µl, mean miR21 Ct values decrease with increasing plasma sample input volume up to 1000 µl. In subsequent EV isolations, 30 µl of SEI reagent and 500 µl of plasma were used.

### RNase-resistant EV-associated miRNA

**Figure 4.**
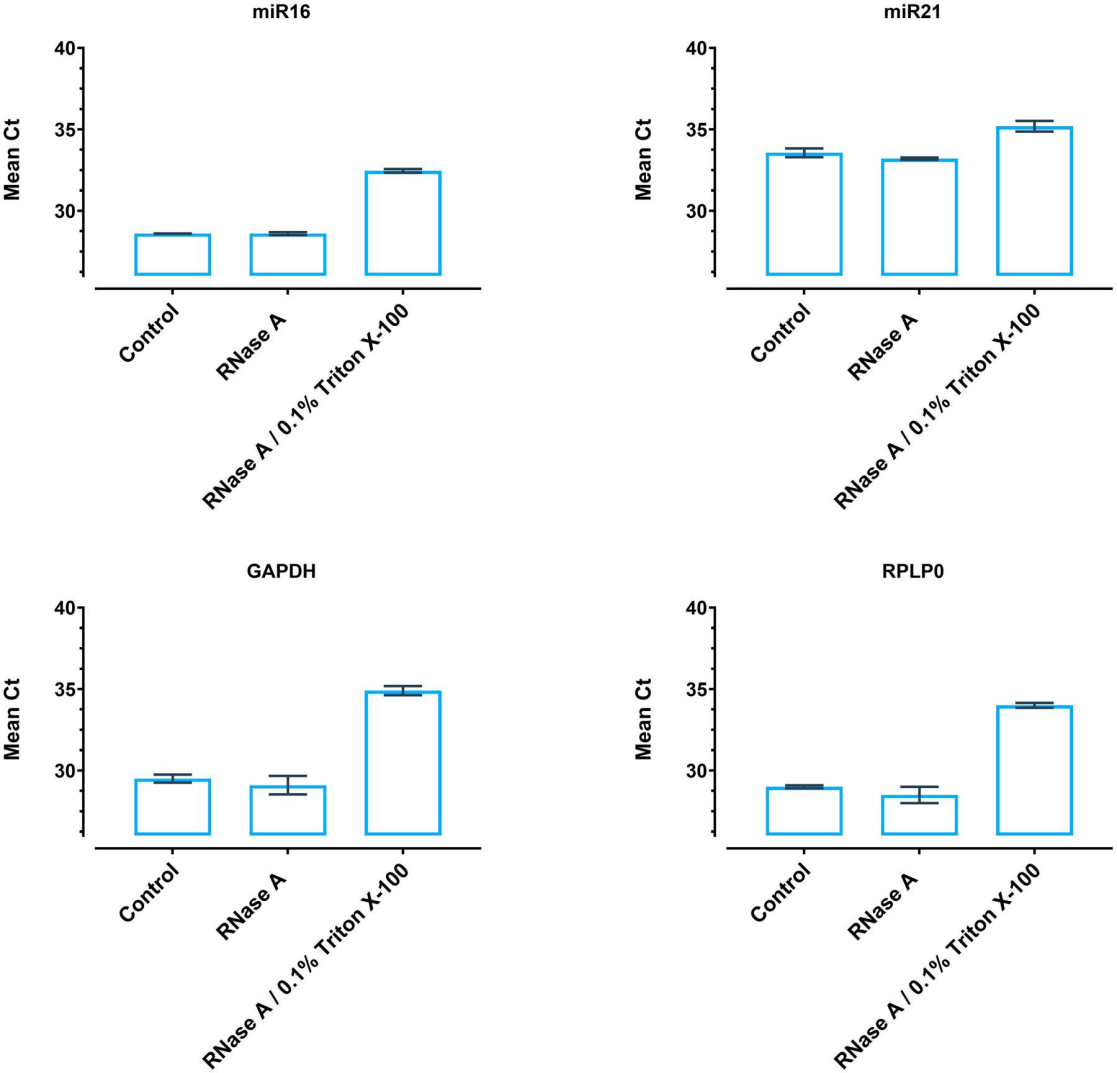
SEI-captured EVs were subjected to RNA extraction followed by RT-qPCR using mRNAs (GAPDH and RPLP0) and miRNAs (miR16 and miR21) specific primers. SEI captured EVs was treated with RNase A (6.25 μg/ml) or RNase A (6.25 μg/ml) with 0.1% Triton X-100. Mean Ct values were not affected by RNase A treatment alone, but degradation was observed following membrane disruption RNase A with 0.1% Triton X-100 (detergent treatment). The mean Ct counts are plotted. Data are presented and the mean ± SEM (*n* = 3).

The effect of treating SEI bead-bound EVs with RNase or RNase + Triton X-100 treatment on mean CT values for mRNA (GAPDH and RPLP0) and miRNA (miR16 and miR21) are presented in (Fig. 4). RNase treatment alone was ineffective in degrading bead-associated RNA and miRNA, as indicated by unaltered Ct values. In the presence of a membrane solubilizing detergent (Triton-X 100), RNase treatment was associated with a significant increase in the Ct values for both mRNA and miRNA transcripts. These data are consistent with the encapsulation of mRNA and miRNA transcripts within an EV.

### Characterization of SEI EVs

#### Protein profile of SEI captured EVs

**Figure 5.**
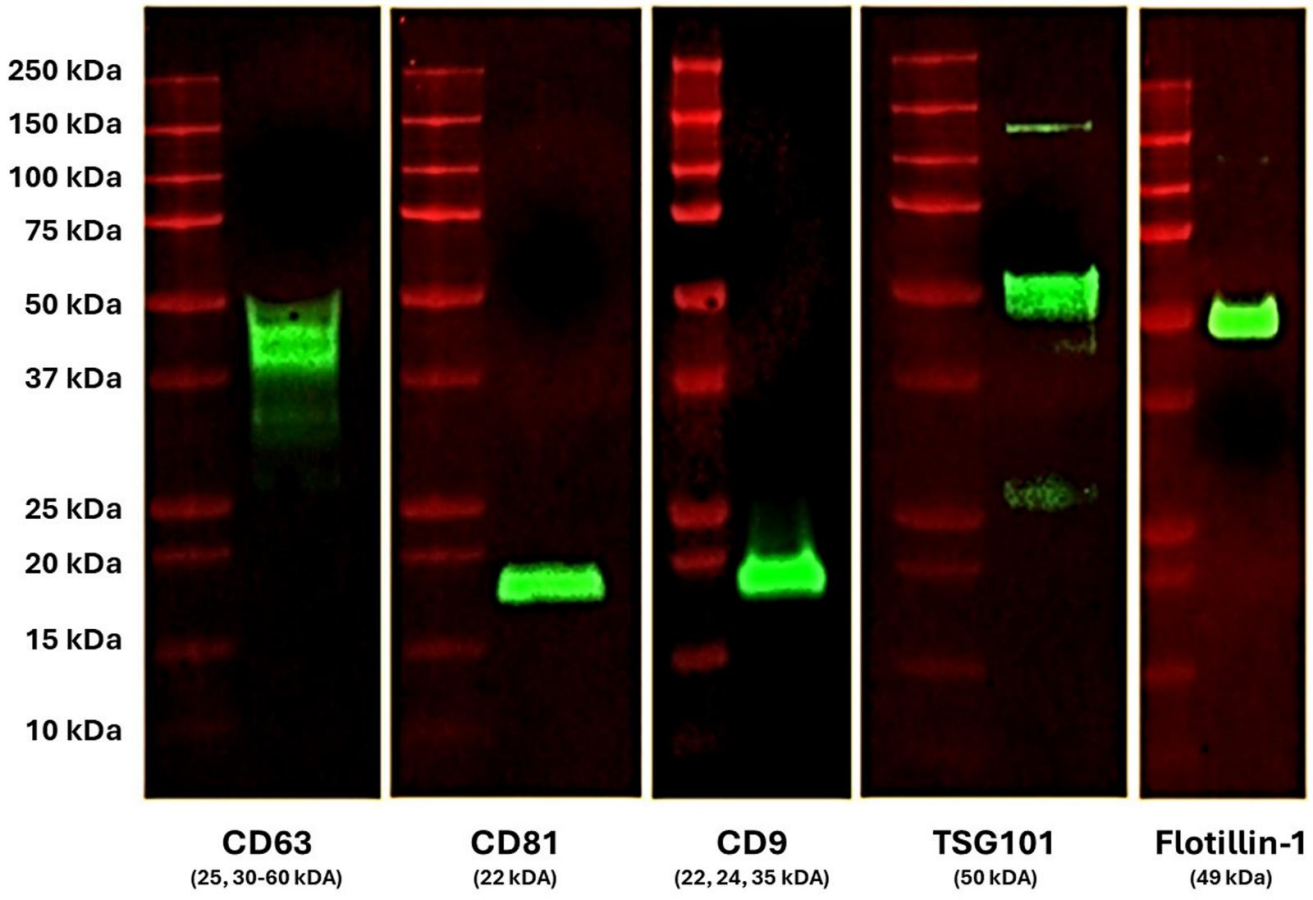
Western blot analysis of SEI EVs isolated from human plasma. EVs capture by SEI from normal human plasma were subjected to on-bead-lysis and Western Blot analysis. EV lysates were positive for CD63, CD81, CD9, TSG101, and Flotillin-1. Data are representative of three independent experiments using pooled normal human plasma.

SEI-captured EVs from plasma samples were then analysed by Western blot for the presence of EVs known protein markers. Western blot analysis confirmed the presence of EVs markers such as CD9, CD63, CD81, TSG101, and Flotillin-1 (Fig. 5) which is in alignment with MISEV2018 guidelines.

### High-throughput automated EV RNA and protein isolation and characterization

**Figure 6.**
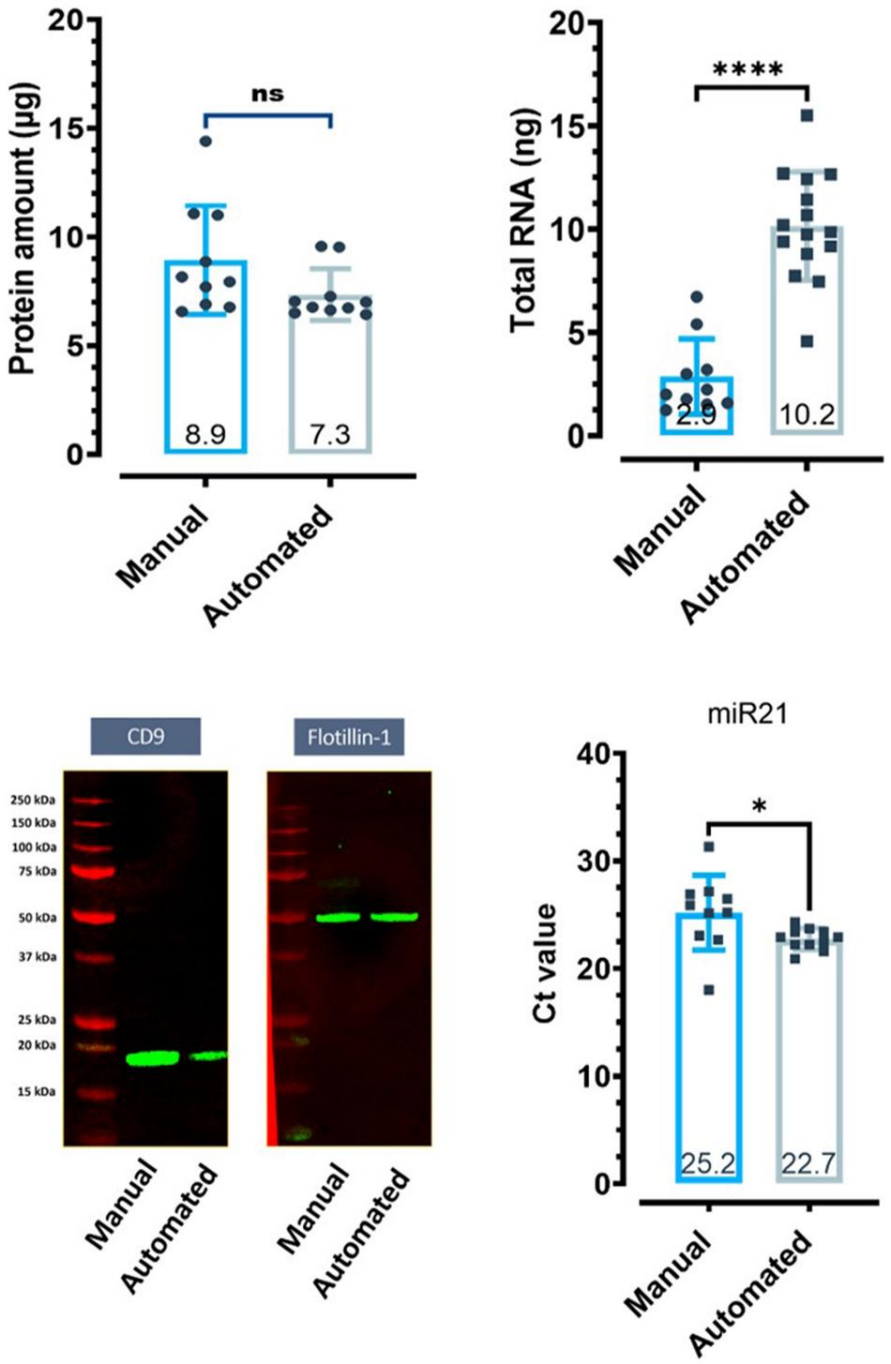
Comparison of manual versus automated EV SEI isolation for the recovery of EV-associated total protein and RNA; the presence of EV markers CD9 and Flotillin-1; and Ct values for EV-associated miR21. Data are presented as the mean and SEM (*n* = 10–15 technical replicates). The western blot is a representative gel. Between group statistical significance was assess by paired Student’s t-tests *P < .05, ***P < .001.

High-throughput SEI EV isolation and EV-associated RNA and protein extraction were established on an automated high-throughput particle moving instrument (KingFisher Apex, Thermo Fisher Scientific). The high-throughput EV RNA and protein yield, and recovery were equal or higher compared to manual processing (Fig. 6). Western blot analysis confirmed the expression of CD9 and Flotillin-1 from isolated EV from both manual and automated systems (Fig. 6). Total RNA yield analysis showed a significantly higher yield of RNA on the automated system than for manual isolation. RT-PCR analysis showed higher expression of miRNAs (miR-16 and miR-21) on the high-throughput automated system than for manual isolation. SEI isolation of EVs was also implemented on the Maxwell^®^ RSC instrument (Promega) for medium-throughput sample processing. The SEI-captured EV-derived miRNA yield and recovery were similar on both instruments (Supplementary Fig. S1) which confirms the compatibility of SEI for EV isolation on automated systems.

#### SWATH analysis

Information-dependent acquisition (IDA) and SWATH profiles were generated from circulating EVs present in clinical samples. The IDA library was used to identify peptide ions that were present in SWATH ion profiles. Proteins were identified and quantified by comparing SWATH-generated peptide ion profiles for each individual sample against the IDA library (PeakView). The total library size used for this project consists of a total of 1748 proteins, of which 1522 proteins were profiled. The variation in the relative abundance of the top 100 EV proteins across the group was established by comparison with the SWATH profile against the IDA library and presented as clustering heat map (Supplementary Fig. S2).

To investigate the potential functions of EV-associated proteins and miRNAs, these molecules were arbitrarily clustered into three groups and each group was subjected to gene ontology analysis. The network included miRNAs and proteins associated with functions related to regulation of cholesterol biosynthetic process, ER to Golgi vesicle-mediated transport, vesicle-mediated transport, and intermediate filament-based process. These data are consistent with the hypothesis that proteins and miRNAs with EV isolated using the SEI method are involved in the trafficking and secretion of EVs.

### miRNA profile of SEI-captured EVs

#### miRNA sequencing analysis

A total of 1476 mature miRNAs species (−5p/−3p) were analysed across the samples, in which 402 miRNA species were detected, defined as having a miRNA count greater or equal to 1. The overall microRNA profile of the top 100 miRNAs across the samples is presented as heat map (Supplementary Fig. S3). Interestingly, two-thirds of the top abundant miRNAs across the samples were associated with vesicle gene ontologies.

#### Comparison with other EV isolation methods

**Figure 7.**
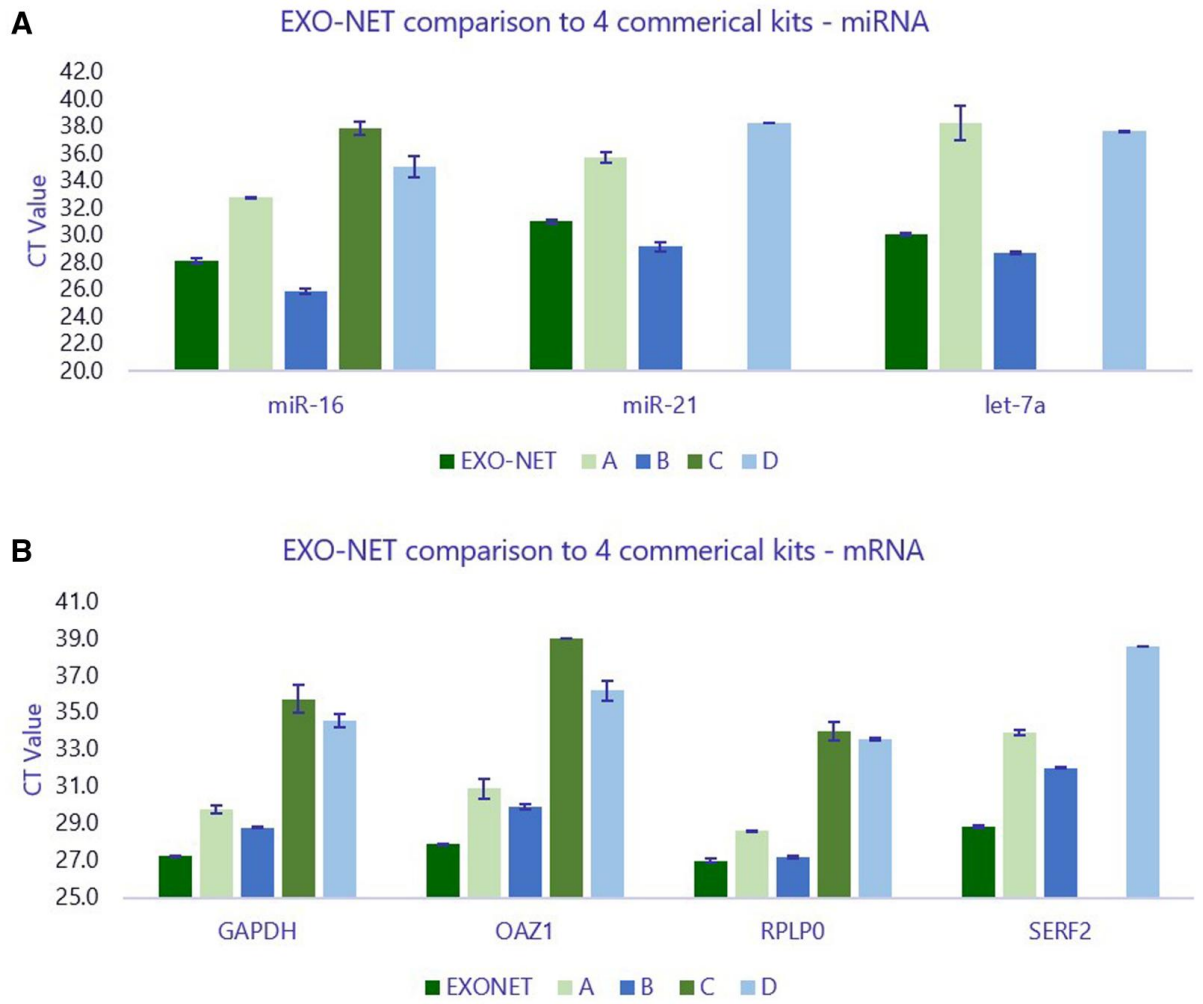
Summary of miRNAs (A) and mRNAs (B) quantification using SEI and four other commercially available kits. EVs were isolated from six individual normal human plasma samples (500 μl each) by SEI and four other exosome isolation kits [A: ExoQuick (SBI); B: ExoEasy (Qiagen); C: Norgen Biotek; D: ThermoFisher kit]. The captured EVs were subjected to RNA extraction followed by RT-qPCR using mRNAs (GAPDH, OAZ1, RPLP0, and SERF2) and microRNAs (miR-16, let-7a and miR-21) specific primers (*n* = 6).

The performance of the SEI reagent was compared to four other EV isolation kits. The outcome measure of performance was the recovery of mRNA (GAPDH, OAZ1, RPLP0, and SERF2) and miRNA (miR-16, let-7a, and miR-21), as determined by RT-qPCR. The expression of miR-16, let-7a, and miR-21 was higher in SEI (EXO-NET) compared to all the kits except the Qiagen’s kit (Fig. 7A); however, there were no significant differences in expression of these miRNAs between EXO-NET and Qiagen kit. Similarly, expression of mRNAs including GAPDH, OAZ1, RPLP0, and SERF2 were higher in EXO-NET-captured EVs compared to the other four EVs isolation kits (Fig. 7B).

## Discussion

Various methods and technologies, including density gradient ultracentrifugation, size-exclusion chromatography, and ultrafiltration, have been employed to isolate EVs based on their physicochemical properties, such as particle density, size, or charge [33, 48]. It is axiomatic that these methods are confounded by the co-isolation of non-EV particles that share similar characteristics that potentially compromise downstream analytical and functional investigations [33]. To address the challenge of co-isolation and minimize contamination by non-EV substances, the implementation of multiple serial isolation processes has been proposed [49]. While such approaches may enhance the enrichment of EVs, they also result in procedural losses, prolonged isolation time, residual contaminants, and alterations in the physical and functional properties of the EVs [50].

The aim of the study was to characterize and compare a SEI EV isolation method that immobilizes EVs on paramagnetic beads for either on-bead-analysis or on-bead-lysis and downstream protein and RNA profiling.

When used in a 96-well plate fully automated format, SEI beads (30 µl/500 µl plasma) recovered an average of 9 µg protein and 10 ng total RNA, with an average sample processing time of <2 min/sample. EV-associated proteins (CD9, CD63, CD81, Flotillin 1, and TSG101 [51, 52]) and RNA (miR16 [53], miR21 [54], GAPDH [55], SERF2 [56], OAZ1 [56], and RPLP0 [57]) were identified in SEI isolates. The mRNA and miRNA captured by SEI beads were resistant to RNase treatment alone but degraded in the presence of RNase and lipid membrane solubilizing detergent. These data are consistent with the encapsulation of nucleic acid within lipid membrane-bound vesicles.

Total protein isolated from 500 µl of plasma was sufficient for proteomic profiling with more than 1500 proteins identified. Utilizing a combined library allowed us to profile over 1500 proteins. Among them, EV-enriched proteins such as CD9 and Flotillin-1 were identified. In addition, SEI-captured EVs had less co-contamination with albumin than the other bead-based EV isolation kits.

Using SEI isolate and small RNA next-generation sequencing, a total of 402 miRNA species associated with human plasma EVs were identified. Of the top 10 abundant miRNAs identified, three have been reported to be highly expressed in blood-derived EVs [58–61, 62]. These miRNAs included: hsa-miR-486-5p, hsa-miR-451a, and hsa-miR-16-5p. hsa-miR-486-5p is highly expressed in muscle, erythrocytes, in an argonaute complex circulating freely within blood and enriched in blood-derived EVs [63]. hsa-miR-451a is abundant in erythrocytes and serum-derived EVs, where it regulates immune response to whole-virus vaccines [64]. hsa-miR-16-5p, which is also enriched in erythrocytes and plasma EVs, has been used to discriminate between oesophageal adenocarcinoma patients and healthy controls within a 5-miRNA biomarker panel [65]. These previous studies, however, used precipitation techniques to isolate EVs, in contrast to our study using SEI technology to provide a miRNA profile of immunocaptured plasma EVs. This approach builds upon the previous findings and enhances our understanding of miRNA profiles within blood-derived EVs.

High-throughput EV isolation and downstream analysis methods that are compatible for translation of EV diagnostics into routine clinical laboratory tests remain limited. In this study, SEI-captured EVs and downstream analysis of associated biomarkers were automated on two platforms, both of which are compatible with routine pathology workflows: the Maxwell RSC and KingFisher Apex. The RT-qPCR analysis showed that SEI-isolation of miRNAs with the Maxwell RSC miRNA Plasma and serum kit was ≥100 times higher than the manual ReliaPrep RNA Cell Miniprep System. Furthermore, using 96-well plate particle moving platforms, an average sample processing time of <2 min/sample can be achieved for the isolation of EV-associated biomarkers. These data confirm that SEI represents a viable solution to solving the main hurdle of screening and isolating EVs from ≥500 samples daily for biomarker discovery and diagnostic applications.

The data obtained in this study demonstrate that SEI is a rapid and simple method to isolate EVs for various downstream analyses, delivering a preparation with greater enrichment of EV proteins and low levels of contamination by high abundance plasma proteins. The yield of EV-associated RNA transcripts is equivalent or superior to other commercially available products. SEI beads can be constructed to capture a diverse range of biological targets, including viral particles, small and large EVs, apoptotic bodies and cells. The effectiveness of SEI methods to enrich EV subpopulations is determined by the specificity and expression of the targeted surface epitopes; most commonly tetraspanins, including CD9, CD63, and CD81 (individually or in combination). Tetraspanins, however, display significant variation in their EV surface expression or may be non-detectable [27]. Targeting additional surface epitopes known to be expressed by specific cell types or under specific physiological or pathological conditions (e.g. NCAM1, CD29, CD146, CD41b, or GAPDH [66–70]) could further enhance the reproducibility and efficiency of EV capture.

Current methods are limited by incompatibility with pathology laboratory workflows, long processing times, and poor yield and purity. Immuno-capture-based methods such as SEI could represent an effective purification alternative to obtain targeted subpopulations of EV samples. Most studies of EV biomarkers are based on EVs that are isolated based on a single feature (e.g. density or size). Such approaches, however, lack specificity and poorly differentiate subpopulations of EVs. To enrich and interrogate subpopulations of EVs, the use of solid-phase EV-surface-specific ligands represents a feasible approach. Here, we present a novel bead-based immunoaffinity system that captures a highly enriched subpopulation of EVs. SEI (EXO-NET) is a magnetic bead-based immunoaffinity EV capture device, where a 3D antibody matrix is constructed on a carbon nanoparticle. The sequential construction of the 3D matrix increases the density and accessibility of ligand binding sites, thereby, increasing the efficiency. The complement of antibodies attached to the bead has been designed to capture a wide range of EVs from different cell types (pan-EV capture). It is used to isolate EVs in solution and allow the downstream analysis of their associated cargo including proteins, oligonucleotides, lipids, and metabolites. SEI products are readily translatable formats for the development and implementation of fully automated applications to be used in routine pathology service laboratories (e.g. FACs, solution array, and ELISA). SEI also can be tuned to preferentially isolate EVs from specific cell types. SEI can provide seamless transition from research and development (where 10–100 s of samples may be processed) to CLIA laboratory workflows (where 100–1000 s of samples are processed). A result of the high avidity of the capture ligands used for SEI is that the release of EVs for functional analysis is limited. The capture and release of intact EVs from SEI for functional analysis is currently under investigation.

## Supplementary Material

bpae032_Supplementary_Data

## References

[1] Aswad H , Forterre A, Wiklander OPB et al Exosomes participate in the alteration of muscle homeostasis during lipid-induced insulin resistance in mice. Diabetologia 2014;57:2155–64. 25073444 10.1007/s00125-014-3337-2 PMC4153976

[2] Desdin-Mico G , Mittelbrunn M. Role of exosomes in the protection of cellular homeostasis. Cell Adh Migr 2017;11:127–34. 27875097 10.1080/19336918.2016.1251000 PMC5351736

[3] Salomon C , Rice GE. Role of exosomes in placental homeostasis and pregnancy disorders. Prog Mol Biol Transl Sci 2017;145:163–79. 28110750 10.1016/bs.pmbts.2016.12.006

[4] Thery C. Exosomes: secreted vesicles and intercellular communications. F1000 Biol Rep 2011;3:15. 21876726 10.3410/B3-15 PMC3155154

[5] Record M. Intercellular communication by exosomes in placenta: a possible role in cell fusion? Placenta 2014;35:297–302. 24661568 10.1016/j.placenta.2014.02.009

[6] Nair S , Salomon C. Extracellular vesicles as critical mediators of maternal-fetal communication during pregnancy and their potential role in maternal metabolism. Placenta 2020;98:60–8. 33039033 10.1016/j.placenta.2020.06.011

[7] Salomon C , Das S, Erdbrugger U et al Extracellular vesicles and their emerging roles as cellular messengers in endocrinology: an endocrine society scientific statement. Endocr Rev 2022;43:441–68. 35552682 10.1210/endrev/bnac009 PMC10686249

[8] Alderton GK. Metastasis. Exosomes drive premetastatic niche formation. Nat Rev Cancer 2012;12:447. 22722393 10.1038/nrc3304

[9] Record M , Subra C, Silvente-Poirot S, Poirot M. Exosomes as intercellular signalosomes and pharmacological effectors. Biochem Pharmacol 2011;81:1171–82. 21371441 10.1016/j.bcp.2011.02.011

[10] Othman N , Jamal R, Abu N. Cancer-derived exosomes as effectors of key inflammation-related players. Front Immunol 2019;10:2103. 31555295 10.3389/fimmu.2019.02103 PMC6737008

[11] McCready J , Sims JD, Chan D, Jay DG. Secretion of extracellular hsp90alpha via exosomes increases cancer cell motility: a role for plasminogen activation. BMC Cancer 2010;10:294. 20553606 10.1186/1471-2407-10-294 PMC3087318

[12] Yoshimura A , Sawada K, Nakamura K et al Exosomal miR-99a-5p is elevated in sera of ovarian cancer patients and promotes cancer cell invasion by increasing fibronectin and vitronectin expression in neighboring peritoneal mesothelial cells. BMC Cancer 2018;18:1065. 30396333 10.1186/s12885-018-4974-5 PMC6217763

[13] Liu J , Wang SZ, Wang QL et al Gestational diabetes mellitus is associated with changes in the concentration and bioactivity of placental exosomes in the maternal circulation across gestation. Eur Rev Med Pharmacol Sci 2018;26:3797–2043. 10.26355/eurrev_202206_28944 35731046

[14] Salomon C , Scholz-Romero K, Sarker S et al Gestational diabetes mellitus is associated with changes in the concentration and bioactivity of placenta-derived exosomes in maternal circulation across gestation. Diabetes 2016;65:598–609. 26718504 10.2337/db15-0966

[15] Sarker S , Scholz-Romero K, Perez A et al Placenta-derived exosomes continuously increase in maternal circulation over the first trimester of pregnancy. J Transl Med 2014;12:204. 25104112 10.1186/1479-5876-12-204 PMC4283151

[16] Caby MP , Lankar D, Vincendeau-Scherrer C et al Exosomal-like vesicles are present in human blood plasma. Int Immunol 2005;17:879–87. 15908444 10.1093/intimm/dxh267

[17] Pironti G , Strachan RT, Abraham D et al Circulating exosomes induced by cardiac pressure overload contain functional angiotensin II type 1 receptors. Circulation 2015;131:2120–30. 25995315 10.1161/CIRCULATIONAHA.115.015687 PMC4470842

[18] Theodoraki MN , Hofmann L, Huber D et al Plasma-derived CD16 exosomes and peripheral blood monocytes as correlating biomarkers in head and neck cancer. Oncol Lett 2023;25:200. 37113401 10.3892/ol.2023.13786 PMC10126691

[19] Ogawa Y , Kanai-Azuma M, Akimoto Y et al Exosome-like vesicles with dipeptidyl peptidase IV in human saliva. Biol Pharm Bull 2008;31:1059–62. 18520029 10.1248/bpb.31.1059

[20] Bachtiar BM , Bachtiar EW, Kusumaningrum A et al Porphyromonas gingivalis association with inflammatory markers and exosomal miRNA-155 in saliva of periodontitis patients with and without diabetes diagnosed with COVID-19. Saudi Dent J 2023;35:61–9. 36540394 10.1016/j.sdentj.2022.12.002 PMC9756571

[21] Chen CY , Hogan MC, Ward CJ. Purification of exosome-like vesicles from urine. Methods Enzymol 2013;524:225–41. 23498743 10.1016/B978-0-12-397945-2.00013-5 PMC4028690

[22] Wu R , Huang C, Wu Q et al Exosomes secreted by urine-derived stem cells improve stress urinary incontinence by promoting repair of pubococcygeus muscle injury in rats. Stem Cell Res Ther 2019;10:80. 30849996 10.1186/s13287-019-1182-4 PMC6408860

[23] Xiang H , Chen S, Zhou J et al Characterization of blood-derived exosomal proteins after exercise. J Int Med Res 2020;48:300060520957541. 32972266 10.1177/0300060520957541 PMC7522842

[24] Zhang Y , Hu YW, Zheng L, Wang Q. Characteristics and roles of exosomes in cardiovascular disease. DNA Cell Biol 2017;36:202–11. 28112546 10.1089/dna.2016.3496

[25] Kowal J , Tkach M, Théry C. Biogenesis and secretion of exosomes. Curr Opin Cell Biol 2014;29:116–25. 24959705 10.1016/j.ceb.2014.05.004

[26] Valadi H , Ekstrom K, Bossios A et al Exosome-mediated transfer of mRNAs and microRNAs is a novel mechanism of genetic exchange between cells. Nat Cell Biol 2007;9:654–9. 17486113 10.1038/ncb1596

[27] Garcia-Martin R , Brandao BB, Thomou T et al Tissue differences in the exosomal/small extracellular vesicle proteome and their potential as indicators of altered tissue metabolism. Cell Rep 2022;38:110277. 35045290 10.1016/j.celrep.2021.110277 PMC8867597

[28] Chen TY , Gonzalez-Kozlova E, Soleymani T et al Extracellular vesicles carry distinct proteo-transcriptomic signatures that are different from their cancer cell of origin. iScience 2022;25:104414. 35663013 10.1016/j.isci.2022.104414 PMC9157216

[29] Puhm F , Flamand L, Boilard E. Platelet extracellular vesicles in COVID-19: potential markers and makers. J Leukoc Biol 2022;111:63–74. 34730839 10.1002/JLB.3MIR0221-100R PMC8667644

[30] Yi Y , Wu M, Zeng H et al Tumor-derived exosomal non-coding RNAs: the emerging mechanisms and potential clinical applications in breast cancer. Front Oncol 2021;11:738945. 34707990 10.3389/fonc.2021.738945 PMC8544822

[31] Kim S , Choi MC, Jeong JY et al Serum exosomal miRNA-145 and miRNA-200c as promising biomarkers for preoperative diagnosis of ovarian carcinomas. J Cancer 2019;10:1958–67. 31205555 10.7150/jca.30231 PMC6548168

[32] Tutrone R , Donovan MJ, Torkler P et al Clinical utility of the exosome based ExoDx Prostate(IntelliScore) EPI test in men presenting for initial Biopsy with a PSA 2-10 ng/mL. Prostate Cancer Prostatic Dis 2020;23:607–14. 32382078 10.1038/s41391-020-0237-z PMC7655505

[33] Jeppesen DK , Fenix AM, Franklin JL et al Reassessment of exosome composition. Cell 2019;177:428–45. e418. 30951670 10.1016/j.cell.2019.02.029 PMC6664447

[34] Rice GE , Salomon C. IFPA Joan Hunt Senior Award in Placentology lecture: Extracellular vesicle signalling and pregnancy. Placenta. 2024 Feb 23. 10.1016/j.placenta.2024.02.007 38458919

[35] Stewart T , Ahmadzada T, Amerena-Cowie S et al Differential detection of cancer-derived extracellular vesicles using combined antibody functionalized magnetic beads and infrared spectroscopy. In: *ISEV Conference 2022: 2022.* Paris, France. J Extracell Vesicles 2022.

[36] Khanabdali R , Lui C, Palma C et al EXO-NET enriched salivary extracellular vesicles in periodontitis. In: *ISEV 2023*. Seattle, WA: J Extracell Vesicles 2023. 10.20517/evcna.2023.48 PMC11648426 39697803

[37] Khanabdal R , Lui C, Palma C et al EXO-NET enriched salivary extracellular vesicles in periodontitis. In: *ISEV Annual Meeting* Seattle, WA: J Extracell Vesicles 2023.

[38] Theodoraki MN , Hong CS, Donnenberg VS et al Evaluation of exosome proteins by on-bead flow cytometry. Cytometry A 2021;99:372–81. 33448645 10.1002/cyto.a.24193 PMC9063195

[39] Khanabdali R , Palma P, Nikseresht S et al High-throughput isolation and enrichment of extracellular vesicles using an immunoaffinity magnetic bead-based matrix. In: *ISEV 2023 Annual Meeting: 2023.* Seattle, WA: J Extracell Vesicles 2023.

[40] Thery C , Witwer KW, Aikawa E et al Minimal information for studies of extracellular vesicles 2018 (MISEV2018): a position statement of the International Society for Extracellular Vesicles and update of the MISEV2014 guidelines. J Extracell Vesicles 2018;7:1535750. 30637094 10.1080/20013078.2018.1535750 PMC6322352

[41] Friedländer MR , Mackowiak SD, Li N et al miRDeep2 accurately identifies known and hundreds of novel microRNA genes in seven animal clades. Nucleic Acids Res 2012;40:37–52. 21911355 10.1093/nar/gkr688 PMC3245920

[42] Langmead B , Trapnell C, Pop M, Salzberg SL. Ultrafast and memory-efficient alignment of short DNA sequences to the human genome. Genome Biol 2009;10:R25. 19261174 10.1186/gb-2009-10-3-r25 PMC2690996

[43] Griffiths-Jones S , Grocock RJ, van Dongen S, Bateman A. Enright AJ: miRBase: microRNA sequences, targets and gene nomenclature. Nucleic Acids Res 2006;34:D140–144. 16381832 10.1093/nar/gkj112 PMC1347474

[44] Love MI , Huber W, Anders S. Moderated estimation of fold change and dispersion for RNA-seq data with DESeq2. Genome Biol 2014;15:550. 25516281 10.1186/s13059-014-0550-8 PMC4302049

[45] Ward MD , Ahlquist JS. Maximum Likelihood for Social Science: Strategies for Analysis. Cambridge: Cambridge University Press, 2018, 36.

[46] Benjamini Y , Hochberg Y. Controlling the false discovery rate: a practical and powerful approach to multiple testing. J Royal Stat Soc Ser B 1995;57: 289–300.

[47] Kern F , Fehlmann T, Solomon J et al miEAA 2.0: integrating multi-species microRNA enrichment analysis and workflow management systems. Nucleic Acids Res 2020; 48:W521–8. 32374865 10.1093/nar/gkaa309 PMC7319446

[48] Zhao Z , Wijerathne H, Godwin AK, Soper SA. Isolation and analysis methods of extracellular vesicles (EVs). Extracell Vesicles Circ Nucl Acids 2021;2:80–103. 34414401 10.20517/evcna.2021.07 PMC8372011

[49] Benedikter BJ , Bouwman FG, Vajen T et al Ultrafiltration combined with size exclusion chromatography efficiently isolates extracellular vesicles from cell culture media for compositional and functional studies. Sci Rep 2017;7:15297. 29127410 10.1038/s41598-017-15717-7 PMC5681555

[50] Jeon H , Kang SK, Lee MS. Effects of different separation methods on the physical and functional properties of extracellular vesicles. PLoS One 2020;15:e0235793. 32634162 10.1371/journal.pone.0235793 PMC7340315

[51] Karimi N , Dalirfardouei R, Dias T et al Tetraspanins distinguish separate extracellular vesicle subpopulations in human serum and plasma—Contributions of platelet extracellular vesicles in plasma samples. J Extracell Vesicles 2022;11:e12213. 35524458 10.1002/jev2.12213 PMC9077141

[52] Koritzinsky EH , Street JM, Chari RR et al Circadian variation in the release of small extracellular vesicles can be normalized by vesicle number or TSG101. Am J Physiol Renal Physiol 2019;317:F1098–110. 31390267 10.1152/ajprenal.00568.2017 PMC6879938

[53] Lange T , Stracke S, Rettig R et al Identification of miR-16 as an endogenous reference gene for the normalization of urinary exosomal miRNA expression data from CKD patients. PLoS One 2017;12:e0183435. 28859135 10.1371/journal.pone.0183435 PMC5578666

[54] Pu C , Huang H, Wang Z et al Extracellular vesicle-associated mir-21 and mir-144 are markedly elevated in serum of patients with hepatocellular carcinoma. Front Physiol 2018;9:930. 30065664 10.3389/fphys.2018.00930 PMC6056643

[55] Jella KK , Yu L, Yue Q et al Exosomal GAPDH from proximal tubule cells regulate ENaC activity. PLoS One 2016;11:e0165763. 27802315 10.1371/journal.pone.0165763 PMC5089749

[56] Dai Y , Cao Y, Kohler J et al Unbiased RNA-Seq-driven identification and validation of reference genes for quantitative RT-PCR analyses of pooled cancer exosomes. BMC Genomics 2021;22:27. 33407103 10.1186/s12864-020-07318-y PMC7789813

[57] Gockert M , Schmid M, Jakobsen L et al Rapid factor depletion highlights intricacies of nucleoplasmic RNA degradation. Nucleic Acids Res 2022;50:1583–600. 35048984 10.1093/nar/gkac001 PMC8860595

[58] Huang X , Yuan T, Liang M et al Exosomal miR-1290 and miR-375 as prognostic markers in castration-resistant prostate cancer. Eur Urol 2015;67:33–41. 25129854 10.1016/j.eururo.2014.07.035 PMC4252606

[59] Yuan T , Huang X, Dittmar RL et al eRNA: a graphic user interface-based tool optimized for large data analysis from high-throughput RNA sequencing. BMC Genomics 2014;15:176. 24593312 10.1186/1471-2164-15-176 PMC4029068

[60] Huang X , Yuan T, Tschannen M et al Characterization of human plasma-derived exosomal RNAs by deep sequencing. BMC Genomics 2013;14:319. 23663360 10.1186/1471-2164-14-319 PMC3653748

[61] Kangas R , Törmäkangas T, Fey V et al Aging and serum exomiR content in women-effects of estrogenic hormone replacement therapy. Sci Rep 2017;7:42702. 28195143 10.1038/srep42702 PMC5307383

[62] Liu T , Zhang Q, Zhang J et al EVmiRNA: a database of miRNA profiling in extracellular vesicles. Nucleic Acids Res 2019;47:D89–d93. 30335161 10.1093/nar/gky985 PMC6323938

[63] Douvris A , Viñas J, Burns KD. miRNA-486-5p: signaling targets and role in non-malignant disease. Cell Mol Life Sci 2022;79:376. 35731367 10.1007/s00018-022-04406-y PMC9217846

[64] Okamoto M , Fukushima Y, Kouwaki T et al MicroRNA-451a in extracellular, blood-resident vesicles attenuates macrophage and dendritic cell responses to influenza whole-virus vaccine. J Biol Chem 2018;293:18585–600. 30282637 10.1074/jbc.RA118.003862 PMC6290151

[65] Chiam K , Mayne GC, Wang T et al Serum outperforms plasma in small extracellular vesicle microRNA biomarker studies of adenocarcinoma of the esophagus. World J Gastroenterol 2020;26:2570–83. 32523312 10.3748/wjg.v26.i20.2570 PMC7265139

[66] Ekstrom K , Crescitelli R, Petursson HI et al Characterization of surface markers on extracellular vesicles isolated from lymphatic exudate from patients with breast cancer. BMC Cancer 2022;22:50. 35012489 10.1186/s12885-021-08870-w PMC8744234

[67] You Y , Muraoka S, Jedrychowski MP et al Human neural cell type-specific extracellular vesicle proteome defines disease-related molecules associated with activated astrocytes in Alzheimer's disease brain. J Extracell Vesicles 2022;11:e12183. 35029059 10.1002/jev2.12183 PMC8758831

[68] Dar GH , Mendes CC, Kuan W-L et al GAPDH controls extracellular vesicle biogenesis and enhances the therapeutic potential of EV mediated siRNA delivery to the brain. Nat Commun 2021;12:6666. 34795295 10.1038/s41467-021-27056-3 PMC8602309

[69] Das P , Mukherjee A, Adak S. Glyceraldehyde-3-phosphate dehydrogenase present in extracellular vesicles from Leishmania major suppresses host TNF-alpha expression. J Biol Chem 2021;297:101198. 34534548 10.1016/j.jbc.2021.101198 PMC8502904

[70] Belov L , Matic KJ, Hallal S et al Extensive surface protein profiles of extracellular vesicles from cancer cells may provide diagnostic signatures from blood samples. J Extracell Vesicles 2016;5:25355. 27086589 10.3402/jev.v5.25355 PMC4834364

