# Supplementary material for: High-throughput surface epitope immunoaffinity isolation of extracellular vesicles and downstream analysis
Source: Biol Methods Protoc. 2024 May 17;9(1):bpae032. doi: 10.1093/biomethods/bpae032 (PMC11272960; doi:10.1093/biomethods/bpae032)
Supplement: bpae032_Supplementary_Data [file bpae032_supplementary_data.pdf]

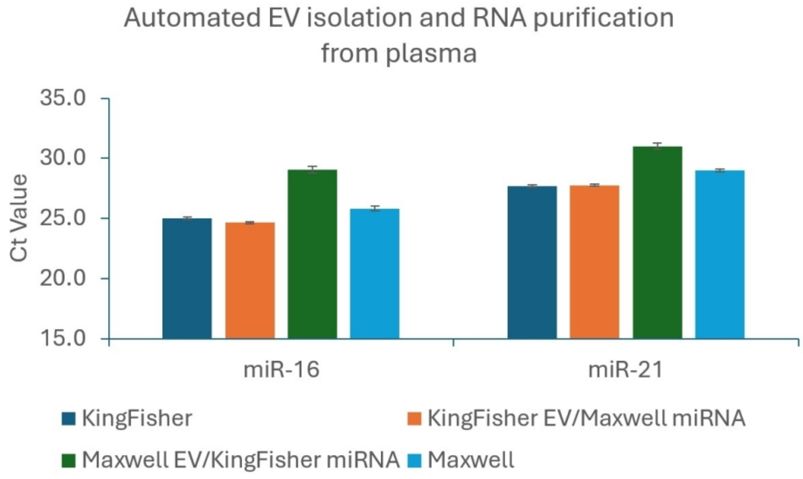

Supp Figure 1: Automated SEI EV and miRNA isolation by mid-throughput Maxwell and high-throughput KingFisher systems. EV were isolated from normal human plasma by SEI on Maxwell and KingFisher instruments. These two instruments were used to purify total RNA from captured EVs using automated Maxwell miRNA Plasma and Serum kit. The SEI EV derived miRNA yield and recovery were similar on both instruments which confirms the compatibility of SEI for EV isolation on automated systems (N=4).

257x140mm (144 x 144 DPI)

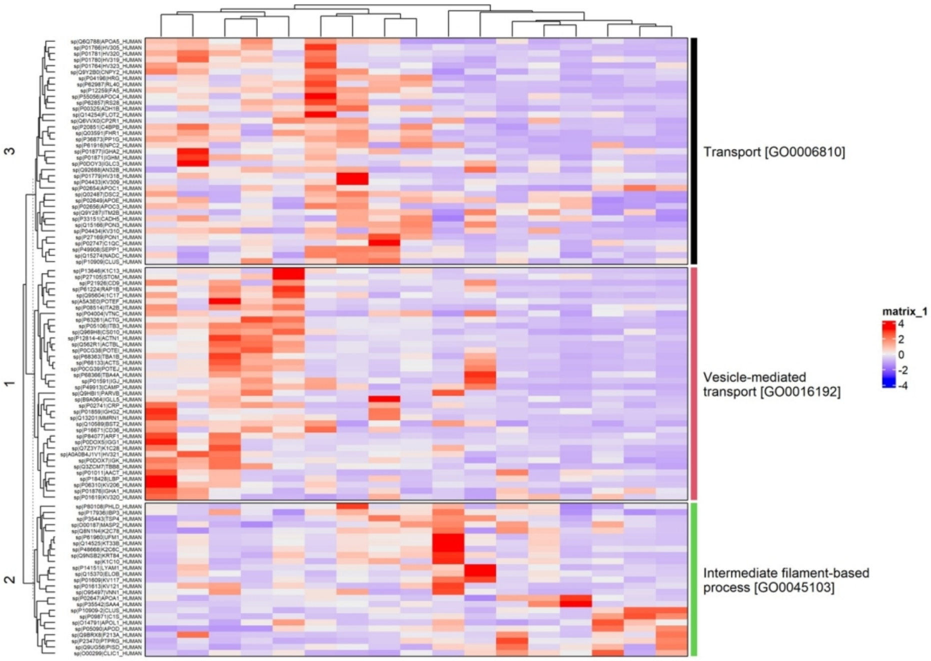

Supp Figure 2: Proteomic profile of SEI captured EVs from normal human plasma. Clustering analysis of the top 100 proteins with the highest medians values across samples were selected. The proteins were arbitrarily clustered into 3 groups, and each group was subjected to gene ontology analysis using Panther (http://www.pantherdb.org/).

451x305mm (96 x 96 DPI)

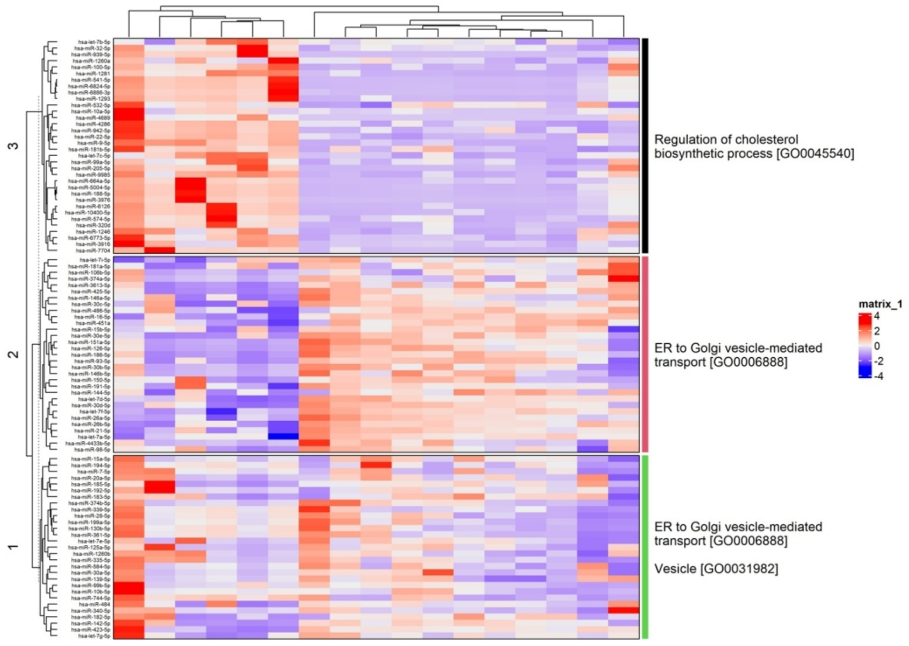

Supp Figure 3: microRNA profile of SEI captured EVs in human plasma. Clustering analysis of the top 100 microRNAs with the highest medians values across plasma samples were selected. The microRNAs were arbitrarily clustered into 3 groups, and each group was subjected to gene ontology analysis using miEAA 2.0 (https://ccb-compute2.cs.uni-saarland.de/mieaa2/).

451x306mm (96 x 96 DPI)
